# Protocol for a Mixed-Method Investigation of the Impact of the COVID-19 Pandemic and Gambling Practices, Experiences and Marketing in the UK: The “Betting and Gaming COVID-19 Impact Study”

**DOI:** 10.3390/ijerph17228449

**Published:** 2020-11-15

**Authors:** Kate Hunt, Nathan Critchlow, Ashley Brown, Christopher Bunn, Fiona Dobbie, Craig Donnachie, Cindy M. Gray, Richard Purves, Gerda Reith, Martine Stead, Danielle Mitchell, Heather Wardle

**Affiliations:** 1 Institute for Social Marketing and Health, University of Stirling, Stirling FK9 4LA, UK; nathan.critchlow@stir.ac.uk (N.C.); a.l.brown@stir.ac.uk (A.B.); r.i.purves@stir.ac.uk (R.P.); martine.stead@stir.ac.uk (M.S.); danielle.mitchell1@stir.ac.uk (D.M.); 2 School of Social and Political Sciences, University of Glasgow, Glasgow G12 8QQ, UK; Christopher.bunn@glasgow.ac.uk (C.B.); craig.donnachie@glasgow.ac.uk (C.D.); Cindy.Gray@glasgow.ac.uk (C.M.G.); Gerda.Reith@glasgow.ac.uk (G.R.); heather.wardle@glasgow.ac.uk (H.W.); 3 Usher Institute, University of Edinburgh, Edinburgh EH8 9YL, UK; fiona.dobbie@ed.ac.uk

**Keywords:** COVID-19, gambling, young adults, commercial determinants of health, sports bettors

## Abstract

The COVID-19 pandemic led to unprecedented restrictions on people’s movements and interactions, as well as the cancellation of major sports events and social activities, directly altering the gambling landscape. There is urgent need to provide regulators, policy makers and treatment providers with evidence on the patterns and context of gambling during COVID-19 and its aftermath. This protocol describes a study addressing the following three questions: (1) How has COVID-19 changed gambling practices and the risk factors for, and experience of, gambling harms? (2) What is the effect of COVID-19 on gambling marketing? (3) How has COVID-19 changed high risk groups’ gambling experiences and practices? This mixed-method study focuses on two groups, namely young adults and sports bettors. In workpackage-1, we will extend an existing longitudinal survey of gambling in young adults (aged 16–24 years) (first wave conducted June–August 2019), adding COVID-19-related questions to the second wave (July–August 2020) and extending to a third wave in 2021; and undertake a survey of sports bettors in the UK (baseline *n* = 4000, ~July–August 2020), with follow-ups in ~October–November 2020 and ~February-March 2021. In workpackage-2, we will examine changes in expenditure on paid-for gambling advertising from January 2019 to July 2021 and undertake a mixed-method content analysis of a random sample of paid-for gambling advertising (*n* ~ 200) and social media marketing (*n* ~ 100) during the initial COVID-19 “lockdown”. Workpackage-3 will involve qualitative interviews with a purposive sample of (a) young adults (aged 18–24 years) and (b) sports bettors.

## 1. Introduction

### 1.1. Background

The World Health Organization’s classification of COVID-19 as a pandemic in March 2020 precipitated unprecedented restrictions on people’s movements and interactions in public and private settings. With the closure of commercial and social venues and the cancellation of major sports events, COVID-19 altered the gambling landscape worldwide. Despite some recent self-imposed limits from the gambling industry on television and radio advertising, the industry continued to promote unaffected products (online slots/casino games, esports, virtual events, all associated with high rates of problem gambling) and incentivise people to start gambling. Additionally, ‘lockdown’ potentially heightens risk factors for gambling and gambling harms (e.g., boredom, stress, anxiety, financial problems, loneliness) [1,2,3]. 

Around 2 million people experience some harms from gambling [4]. Gambling behaviour is variable; people transition in and out of harms [5,6] in response to life experiences and conditions. This, combined with corporate practices to maintain market position and profits (e.g., cross-selling customers into casino games or heavy incentivisation to open accounts), suggests the potential for a heightened risk for gambling harms in the UK following COVID-19 responses. As there is evidence that gambling products are designed to be not only attractive but also addictive [7], habits formed during different stages of COVID-19 “lockdown” may persist once these restrictions lift or change. 

There is an urgent need to provide regulators, policy makers and treatment providers with high-quality evidence on the changing patterns and context of gambling behaviours during COVID-19 and its aftermath. During the initial COVID-19 outbreak, some jurisdictions, though not Britain, acted to protect their populations from gambling harms; for example, Belgium introduced mandatory loss limits, Latvia and Portugal stopped online gambling and Spain temporarily banned marketing and advertising. To inform treatment and support provision, insight is needed into the following: the actions undertaken by industry, so regulators can consider appropriate responses; the potential for new at-risk groups, susceptible to gambling-related harms, to emerge and the prevention strategies that might limit these harms; and the potential escalations or continuations of established harms. 

To date, research on the impact of COVID-19 on gambling is very limited. Others have called for “timely, systematic” research on the potential change in gambling worldwide, and have highlighted how the consequences of social isolation during social distancing, boredom and increased financial pressures may all impact gambling behaviours, citing evidence of increased problem gambling during previous economic and financial crises in Greece and Iceland [8]. Håkansson et al. also raise the possibility that, as a result of COVID-19, “online gambling may constitute a particular health hazard when many people are confined to their homes and have had rapid changes in working conditions, psychological stress, anxiety and depression” [8]. As yet, no clear picture of the impact of COVID-19 on gambling activities and gambling harms has emerged. A cross-sectional online survey of 2005 gamblers during the first six weeks of COVID-19 emergency measures in Ontario, Canada, investigated gambling behaviours and motivations, financial circumstances, mental health and substance use and the influence of COVID-19 on online gambling and suggests that “COVID-19 has had a turbulent effect on the lives of Canadians, including gamblers in Ontario and especially those who play online” [9]. Håkansson conducted a cross-sectional web survey in Sweden with a sample of ~2000 people (51% men) recruited from the web panel of a market survey company. Only a minority of the sample reported a change in their gambling habits, with more reporting a decrease than an increase in gambling. However, those who reported an increase in gambling also reported an increase in alcohol use during the pandemic and “markedly higher gambling problems” [10]. Another study in Sweden analysed data provided by the Swedish Gambling Authority on the number of attempts to initiate gambling sessions per day between 1 January 2020 and 8 April 2020 and reported a decrease in the number compared to those forecast in the period from mid-March to 8 April [11]. Auer and colleagues also investigated short-term impacts of the pandemic on online sports bettors before and after various European governments imposed measures to curtail the spread of COVID-19. They analysed the gambling behaviours of sports bettors in Sweden, Germany, Finland and Norway using data provided by a large European online gambling operator from the beginning of 2020 to April 2020 and reported that the number of sports bettors who were active daily and the amount wagered on sports decreased during March and April 2020 [12].

These early studies reflect widespread concern to understand whether COVID-19 impacts on gambling behaviours, and they mostly report on the analysis of online surveys or other quantitative data over the initial few months of 2020, before and immediately after COVID-19 first began to spread widely in parts of Europe and North America. This protocol paper describes our multi-method approach to studying the impact of COVID-19 and gambling practices, potential harms and marketing over a longer period of time, on people living in the UK, with a focus on young people and people who regularly bet on sports. 

### 1.2. Study Aims and Research Questions

This study aims to address the following three overarching research questions (RQs): (RQ1) How has COVID-19 changed gambling practices and the risk factors for, and experience of, gambling harms? (RQ2) What is the effect of COVID-19 on gambling marketing? (RQ3) How has COVID-19 changed high risk groups’ gambling experiences and practices? We hypothesise that two groups, young adults and sports bettors, may experience particular risk of adopting more risky, online gambling practices during the COVID-19 pandemic and its associated restrictions. Young adults are at high risk of experiencing gambling harms [13], with high incidence rates for problem gambling. They are also a group who may be particularly affected by changes to their ability to socialize (e.g., meeting in licensed premises, meeting outdoors) or their prospects for and conditions of work. Regular sports bettors, immersed in sporting environments where gambling is normalised, are arguably most affected by changes in the gambling landscape following lockdown restrictions put in place to limit the spread of COVID-19 during 2020; and it is anticipated that, as restrictions ease, there will be a proliferation of sports events on which to gamble and likely heightened industry marketing and advertising.

## 2. Materials and Methods: Study Aims, Methods and Design

**Figure 1:**
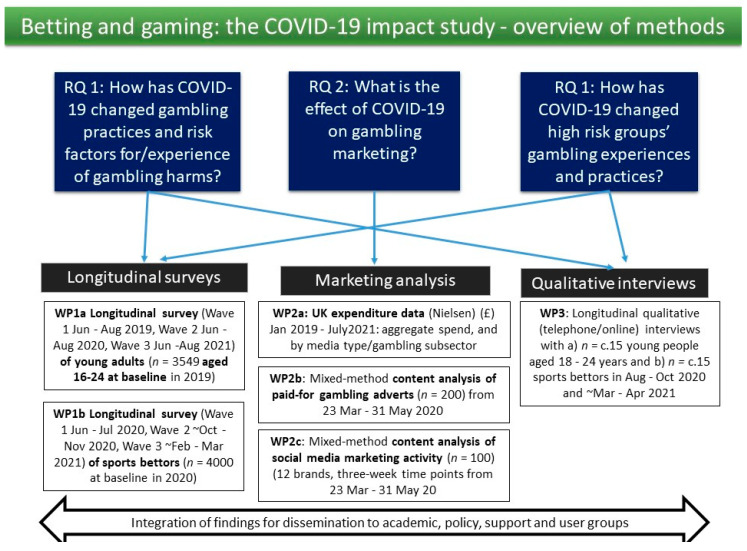
Overview of research questions and methods.

The study will address its three overarching research questions across three integrated workpackages (WPs) (see Figure 1), which are described below.

### 2.1. WP1: How Has COVID-19 Changed Gambling Practices and the Risk Factors for, and Experience of, Gambling Harms?

**Figure 2:**
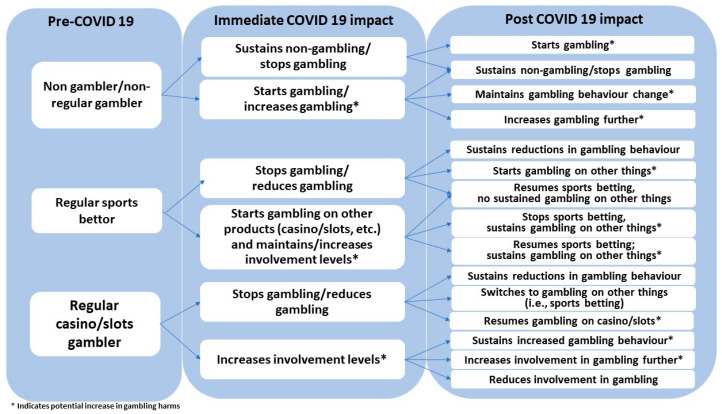
Potential pathways to COVID-19-related changes in gambling behaviours.

We will collect and analyse bespoke longitudinal data for two groups, namely young adults (WP1a) and sports bettors (WP1b), through online surveys. Both studies will examine changes in gambling behaviour; the potential pathways to behaviour change are shown in Figure 2.

WP1a: For the longitudinal surveys of young adults, we will adapt (by adding COVID-19 related questions to a follow-up planned for June–August 2020) and extend (by gathering an additional follow-up in June–August 2021) an ongoing detailed study of gambling behaviours in young adults, aged 16–24 years when first surveyed in 2019. The protocol for this study is available elsewhere [14]. UK-wide data were collected in the year before the emergence of COVID-19 (wave 1, June–August 2019) from 3549 people aged 16–24 sampled from the YouGov panel, and included detailed data on the following: gambling behaviours, problem gambling and experience of gambling harms, social media use, video game use and engagement in gambling-like mechanics within games, esports betting, exposure to gambling advertising and impact, watching live sports events, impulsivity, personal subjective well-being (including anxiety), suicidal thoughts and attempts, loneliness, deprivation and social fragmentation and risky drinking behaviour.

Another wave of data collection, planned and funded prior to the outbreak of the pandemic, will be adapted to include specific questions on the impact of COVID-19 on gambling practices, experiences and potential harms, and on life circumstances, during/immediately after the initial COVID-19 lockdown (wave 2, June–August 2020). Baseline (2019) and 2020 data will thus enable examination of immediate impacts of the pandemic and initial lockdown period in the UK. The second follow-up (wave 3, June–August 2021) will provide evidence on long-term trends.

WP1b: Unlike for young adults, there is no existing research looking in detail at the gambling behaviours of sports bettors in Britain. In summer 2020, we will recruit a longitudinal panel of around 4000 adults (aged 18+) who were regular (at least monthly) sports bettors pre-COVID from a longitudinal panel of regular online bettors (~70,000 people) maintained by YouGov. Sports betting includes betting on any type of live sports events (not esports or virtual races/events) or horse races. Drawing on the survey instruments described above, with an additional suite of questions related to sport and gambling on sport, baseline (wave 1) interviews will be collected during ~July–August 2020, with follow-ups planned in ~October–November 2020 (wave 2, YouGov predicted response 2800/4000, 70% retention) and ~February–March 2021 (wave 3, predicted response 2400/4000, 60% retention). The proposed sample sizes allow us to detect changes in problem gambling rates (assuming a 5% rate at baseline and between wave correlation of 0.5) of ±0.4 pp (at 80% power). This level of power will allow examination of changes in behaviour among subgroups and their relationship with broader changes in economic and social life. The online survey will include measurement of gambling behaviours prior to, and during, the COVID-19 pandemic; experience of problem gambling and harms during the COVID-19 pandemic; motivations to gamble during the COVID-19 pandemic; awareness of, and participation with, gambling advertising/marketing during the COVID-19 pandemic; engagement in watching/attending sports events as they are reintroduced post “lockdown”; changes in other health risk behaviours and emotional well-being during the COVID-19 pandemic; and changes in life circumstances during the COVID-19 pandemic.

These data will enable us to examine whether people adopt any higher risk forms of gambling, such as online casino products, virtual races/events or esports in the short and longer term, the characteristics of those who transition to different forms of gambling and whether these transitions persist, either replacing or continuing alongside known patterns of gambling on live sports events (see Figure 2).

### 2.2. WP2: What Is the Effect of COVID-19 on Gambling Marketing?

To understand the impact of COVID-19 on gambling marketing, we will examine the following: (1) trends in how and where gambling is advertised before/during/after COVID-19; (2) how the pandemic and initial lockdown period in the UK in 2020 influenced what gambling products were heavily advertised; and (3) what messages were promoted about gambling during the initial lockdown period in the UK in 2020 (see Figure 1). 

For WP2a, we will purchase expenditure data on paid-for advertising to examine changes in where gambling is advertised and which subsectors are heavily advertised before/during/after the COVID-19 outbreak in the UK. Weekly data (£) from 1 January 2019 to 31 July 2021 will be purchased from Nielsen, a media monitoring agency which captures data for “traditional” and some digital advertising. Weekly data will include aggregate spend and breakdowns by media type (e.g., press/television) and gambling subsector (e.g., bookmakers/bingo). 

In WP2b, we will explore what messages are communicated in gambling advertising during the COVID-19 pandemic. To achieve this, we will conduct a mixed-method content analysis of paid-for gambling adverts. A stratified random sample of adverts (creatives) (*n* = 200) will be purchased from Nielsen. Adverts will be selected from the week beginning 23 March 2020 to the week ending 31 May 2020, defined as the initial lockdown period and when almost all live sport in the UK was suspended. We intend to use data from WP2a to stratify the random sample by media channel or gambling subsector, to ensure representativeness. Data will be coded using an adapted version of a validated codebook used in a previous study of gambling advertising, conducted on a recent sample of adverts from March 2018 (therefore providing a degree of comparability). This codebook generates empirical and qualitative data on advert characteristics, design features, topical associations, gambles and offers promoted and harm-reduction messages. 

For WP2c, we will conduct a mixed-method content analysis of gambling marketing from Twitter, YouTube, Instagram and Facebook. Data will be collected from the official/verified UK pages of 12 gambling brands. The brands will be purposively selected to represent different subsectors (e.g., bookmakers/online casino), operator type (e.g., land-based/online-only/both), and popularity (based on public market research data) [15]. For each brand, and each platform, posts will be captured from three weeks within the initial lockdown period: 30 March to 5 April 2020 (“early lockdown”), 20–27 April 2020 (“mid-lockdown”) and 11–17 May (“later lockdown”, when some sports (mostly international) resumed). We will only collect the posts made by the brands themselves (i.e., excluding replies to other users/comments by other users). From the collected data, and mirroring WP2b, we will select a random stratified sample of posts to analyse (*n* = 100). To ensure representativeness, stratification and selection of posts will be informed by the proportion of posts collected from each social media platform and the proportion from different gambling subsectors. All selected posts will then be analysed using the same adapted codebook as WP2b. Field notes from the data extraction and sampling processes will also enable consideration of general marketing strategies and activities during the initial COVID-19 lockdown. 

### 2.3. WP3: How Has COVID-19 Changed High Risk Groups’ Gambling Experiences and Practices?

Qualitative interviews will be undertaken at two time points with purposively selected samples of (a) young people aged 18–24 years and (b) people who regularly bet on sport pre-COVID-19. Using multiple channels, including social media (e.g., Facebook, Twitter, online fan groups), gatekeeper and third sector organisations, and snowballing, we will recruit ~30 people (~15 in each group, with sample diversity by sex, age, socio-economic status, etc.) to participate in in-depth interviews in ~July–September 2020, and follow-up interviews in ~March–May 2021, i.e., around one year after COVID-19 restrictions were first imposed in the UK. We will attempt to interview at least some of the same participants at both time points, and the follow-up sample may be augmented by recruiting other participants to the study (e.g., if it is not feasible to re-contact participants in the follow-up interviews in ~March–May 2021). 

We aim to recruit young adults aged 18–24 years with some experience of betting or gambling prior to COVID-19 or who have become involved in betting/gambling for the first time during the outbreak and immediate aftermath of COVID-19. Some of the young adults may also be sports bettors. We aim to reflect the population of sports bettors by ensuring that around two-thirds of the sample (*n* ~ 10) are aged 18–40 years and around one-third of the sample aged 40+ years (*n* ~ 5). While it is anticipated that the majority of sports bettors will be male, we will aim to recruit some women to each group. All potential participants will be asked to answer some screening questions (by telephone) prior to taking part (including age, gender, type of gambling activity pre-COVID-19/during COVID-19 and contact details) to assess participant suitability for the interview and ensure our sampling criteria are met. At the end of the interview, structured questions will collect information on ethnicity, highest educational qualification and problem gambling score.

To reflect current COVID-19 restrictions and regulations (e.g., social distancing), interviews will be conducted remotely, e.g., by telephone/Microsoft Teams, according to participant preference. Follow-up interviews will be conducted in the same way as far as possible to ensure consistency. With participant consent, interviews will be audio-recorded (using an encrypted device) and transcribed (intelligent) verbatim. Data will be managed and retrieved using NVivo 12 (QSR International, Melbourne, VIC, Australia) to facilitate robust analysis.

Topic guides will facilitate detailed discussion of specific topics of interest emerging from the surveys (see WP1 above), encouraging people to reflect on whether (and how) their experiences of the COVID-19 outbreak have shaped their day-to-day life, employment, use of time and leisure activities, including their consumption of sport, their gambling practices, their online and interpersonal social interaction and other salient aspects of their lives. Selected marketing materials from WP2 may be used to prompt discussion about responses to various marketing strategies, media and content. 

### 2.4. Ethical Considerations

In our planning of the surveys and qualitative interviewing, we have recognised that gambling can be a sensitive issue and some participants may be concerned about gambling-related harms, but we do not expect there to be undue ethical risks associated with this study. With the exception of a small number of young adults from WP1a survey respondents, in 2020 all participants will be above the minimum legal age for all forms of gambling in the UK (i.e., ≥18 years) and we are not explicitly targeting any highly vulnerable individuals (e.g., by recruiting through agencies supporting people with known problems with gambling harms for the qualitative interviews). All participants will provide explicit written consent to participate at each wave of the longitudinal surveys, and either written consent or audio-recorded verbal consent prior to any qualitative interviews. Prior to taking part, participants will be informed about who is conducting the research, that their involvement is voluntary, and that they have the right to withdraw at any time without penalty. The survey samples will be drawn from existing online YouGov panels, and participants will already be accustomed to completing online surveys (including questions on potentially sensitive issues related to lifestyle and health risk behaviours). 

As part of the YouGov panels, participants receive a small remuneration in the form of loyalty points that can be redeemed for vouchers once certain point thresholds are met. Participants will receive between 50–150 points (equivalent to £0.50 to £1.50) on their YouGov account for completing each survey. Participants in qualitative interviews will be offered a £20 online shopping voucher as a gesture of thanks for their participation.

All survey data will be anonymised by YouGov prior to the secure transfer of data to the research team, who will only have access to a unique anonymous participant ID (i.e., no personal details such as name, address or e-mail address of any survey panel participants). Many of the survey questions will be based on established and validated survey tools; nevertheless, as we recognise some participants may be concerned about answering questions about their gambling behaviour and their experience of gambling harms, the electronic information sheets and consent forms will remind participants that their responses will be completely anonymous and confidential, their participation is voluntary, and they can withdraw at any time without any penalty. The survey participant debrief forms include signposts to sources of information and support about gambling and welfare (e.g., GamCare, The Samaritans); similar signposting will be available to participants in the qualitative interviews. Following the protocols used on the Adult Psychiatric Morbidity Surveys, anyone who reports any suicidal thoughts or behaviours during the surveys will be immediately directed to speak to their GP and given the details of support organisations. Should such an event occur, the research team will inform YouGov of the relevant anonymous participant ID so YouGov can follow their safeguarding protocols.

Prior to obtaining consent for the qualitative interviews, we will ensure all participants are fully informed and understand the nature and purpose of the study. They will be assured that the study is confidential and entirely voluntary. We will also check at the start of each interview that the participant is in a place where s/he feels comfortable about being able to speak freely without being overheard by anyone s/he does not wish to share her/his responses with and agree a form of words they can use to indicate that s/he thinks someone has come into the room or may be able to overhear her/his responses. 

We have sought and gained ethical approval for the study from the University of Stirling General University Ethics Panel, Stirling, UK. The survey measures, and methods of response, will also be reviewed by YouGov’s in-house survey experts, who have leading experience in the delivery of surveys on sensitive topics. The research team has extensive experience of the ethical issues of conducting research on a range of potentially sensitive topics (including gambling, alcohol, tobacco use and weight management) and includes internationally recognised experts in gambling behaviours and gambling harms well versed in, and sensitised to, the potential issues raised by gambling research. 

Our data management plan includes details of storage of data to ensure secure storage.

Ethical review was undertaken and approval granted by the University of Stirling General University Ethic Panel (GUEP), reference numbers GUEP 934 (1.7.20) and GUEP 930 (26.6.20).

### 2.5. Patient and Public Involvement

A Study Steering Committee will include members with experience in (a) working with people to prevent gambling harms (e.g., Betknowmore UK, Beacon Counselling Trust), (b) promoting public health through sport (Healthy Stadia) and (c) those with lived experience of gambling harms. The Steering Group will advise on all aspects of study design, in particular reviewing survey questions, early outputs and topic guides. 

## 3. Analysis

For both WP1a and WP1b, initial analyses will focus on (a) mapping transitions in participants’ behaviours across the three waves of data collection and (b) examining the characteristics of those whose self-reported experience of gambling harms changes between waves. For sports bettors (WP1b), participation in 23 different gambling activities will be collected in each wave. This will include estimations of gambling frequency and time and money spent gambling for each activity. This will be used to develop participant transition matrices, looking at who starts and stops participating in each activity and associated changes in their levels of gambling involvement (frequency, time and money spent). For WP1a, participation in 18 different gambling activities was collected, with frequency of gambling reported for each. Time and money spent gambling were collected in aggregate. WP1a contains slightly less detail on gambling behaviours than WP1b as this is a general population sample, containing far fewer gamblers. For example, in wave 1 (data collected in 2019 [14]) only 101 people reported having gambled in a casino in the past year, rendering this study underpowered to look at detailed engagement in each specific gambling activity. However, there are a large number of non-gamblers in wave 1 (*n* = 2053) meaning that gambling onset in specific activities can be computed.

For both WP1a and WP1b, changes in problem gambling scores, measured using the Problem Gambling Severity Index, will be assessed. We will also examine self-reported gambling harms to assess whether these have remained stable, increased or decreased since the previous interview. From this, we will examine the incidence of problem gambling between waves and subgroups whose problem gambling scores have changed in order to describe their characteristics. An initial analysis plan for WP1b, wave 1 has been pre-registered, and further details about WP1a can be found elsewhere [14]. 

For both studies, both bivariate and multivariate analyses will be conducted. Data will be weighted for non-response between waves. At wave 1, data will be weighted to reflect the age, sex and regional profile of their respective populations (the general population for WP1a; regular sports bettors for WP1b). Analyses will be conducted using SPSS v25 (IBM, Armonk, NY, USA) and Stata Statistical softward, version 15 (StataCorp LP, College Station, TX, USA), using the complex survey commands to take into account the weighted and stratified sampling design.

In WP2a, analyses of advertising spend today will examine the direct impact of restrictions on social movement and suspension of live sport (e.g., March–May 2019 vs. 2020 and 2021) and the longer-term implications of the pandemic and resumption of live sport (e.g., weekly trends in 2019 versus 2020 and 2021). Descriptive and time series analyses will examine changes in aggregate expenditure and changes within and between advertising media and gambling subsector. The WP2a data will also be triangulated with wider parts of the study to examine how trends and changes in gambling among the identified risk groups correspond to changes in advertising spend. For example, we will explore whether greater uptake or spend on casino and poker gambling among sports bettors is also reflected in enhanced spend on advertising for this subsector while gambling spend and activity on live sport were restricted.

In WP2b, we will use the same method and coding approach as a prior study conducted in 2018–2019, to allow efficient comparison of gambling adverts in circulation during the initial COVID-19 lockdown period with advertising that occurred prior to the COVID-19 outbreak. In WP2c, textual and visual data will be analysed using the same codebook developed for WP2b. Further details on the validated codebook and methods of analysis are reported elsewhere [16,17]. We will revise aspects of the codebook to ensure data are gathered on aspects of marketing that may be specifically targeted at sports bettors or young people, for example, analysing to what extent bookmaker marketing promotes virtual sports, esports or real-world events held outside the UK to maintain interest and engagement among sports bettors during the pandemic. 

In WP3, all interviews will be audio-recorded with permission and transcribed by a university-approved transcription company. Each transcript will be checked for accuracy prior to analysis. Each participant will be allocated a unique ID or pseudonym and any other identifiable information will be de-identified where possible. The de-identified versions of each transcript will be used for data management and data analysis. Qualitative data analysis will utilise the framework approach, a robust, structured and systematic method, incorporating inductive and deductive reasoning [18]. We will use a thematic approach to analyse the data, facilitated by NVivo 12. First, we will read the transcripts to identify the key topics and issues that emerge from the data. Next, a draft analytical framework will be created, piloted, refined and finalised by the project team. Each transcript will then be coded and summarised into key themes using Framework matrices or charts. This approach reduces large volumes of data and facilitates systematic between-case and within-case analysis. It also allows for emergent patterns and explanations to be explored and tested and, thus, provides the depth required to move beyond description and into interpretative analysis, which is the aim of qualitative analysis. Use of NVivo 12 ensures that analysis is fully documented and conclusions can be clearly linked back to the original source data.

## 4. Discussion

This project was funded under the UK Research and Innovation (UKRI) call for rapid response research in relation to the COVID-19 pandemic and was designed to respond to an urgent need to provide regulators, policy makers and treatment providers with high-quality evidence on the changing patterns and context of gambling behaviours during the initial outbreak of COVID-19 and related restrictions (“lockdown”, cancellation of sport, etc.) and its aftermath. To inform treatment and support provision, insight is needed into the actions undertaken by the gambling industry, so regulators can consider appropriate actions in response, as well as an understanding of new at-risk groups susceptible to the experience of harms, in order to develop effective prevention strategies, and an understanding of the escalation and maintenance of harms. The COVID-19 pandemic is still unfolding and unlikely to resolve until an effective vaccine is produced, with good uptake internationally. The findings of this research will thus provide useful insights into how various aspects of “lockdown” restrictions on daily life, which may be successfully imposed and eased at local, regional and national levels during 2020–2021, may exacerbate or protect people from the risks of gambling-related harms.

While a comprehensive discussion of all the potential methodological limitations of this large multimodal mixed-method study is beyond the scope of this protocol, we highlight some potential limitations that will be discussed in the reporting of our findings in the future. First, participants from our surveys will be recruited from a non-probability online panel maintained by YouGov. While this approach raises some issues about generalisability, regular sports bettors are a hard-to-reach and niche group, and the YouGov panel has the benefits of allowing us to identify people who were regular (at least monthly) sports bettors *prior to* the COVID-19 pandemic and to sample from that pool. Another advantage of this approach is that it enables us to conduct this COVID-19-related research in a very timely manner, responding to the needs of policy makers, public health specialists and regulatory bodies.

In our analyses of gambling marketing, expenditure data are limited to paid-for advertising (i.e., not social media) and we are only be able to examine advertising activities covered by Nielsen. While the data will provide important insight into overall trends, they are likely to underestimate cumulative marketing spend across all media and all gambling subsectors. Others have drawn attention to the sheer, and increasing [19], volume of gambling marketing on social [20] and more traditional media, and we consider that a detailed understanding of the initial lockdown period will allow unique insights into the impacts of COVID-19. Hence, we have chosen to sample adverts for a detailed analysis of content from this period, but this “deep-dive” analysis will not be able to provide insight into longer-term trends in gambling advertising content (e.g., when some major sporting events began to restart in the UK). We are able, however, to look at longer-term trends through the marketing spend data.

As noted by Vindrola-Padros et al., the COVID-19 outbreak demands “the timely sharing of not only epidemiological data but also research findings related to disease perception, social practices that might be linked to spread, health-seeking behaviors, health care delivery models, and barriers to care”. The authors highlight both the “importance of qualitative data to inform evidence-based public health response” (p. 2192) and the challenges and practical issues raised [21]. In response to the practical issues imposed by restrictions on travel and social interactions, our approach to the qualitative interviews was to ensure that all participants could take part in interviews remotely. The established expertise in our research team in qualitative data collection and analysis, in-depth understanding of existing social science research on gambling (see, e.g., [5,6]), and prior experience of working together as a team has facilitated the timely conduct of the qualitative component of our study. 

## 5. Conclusions

Collecting timely data on the ways in which patterns and experiences of gambling change in key groups of people engaged in betting and gambling, alongside data on marketing spend and approaches, will be essential to informing regulatory bodies and others concerned with the prevention and treatment of gambling harms.
